# “Sickle cell trait and haemophilia: a rare association”

**DOI:** 10.11604/pamj.2018.29.92.14551

**Published:** 2018-01-30

**Authors:** Hayat El Maataoui, Amina Fahi, Bouchra Oukkache

**Affiliations:** 1 Ibn Rochd Teaching Hospital Hematology Laboratory Casablanca, Maroc; 2 Faculty of Medicine and Pharmacy, Casablanca, Maroc

**Keywords:** Haemophilia A, sickle cell trait, sikle cell disease, association

## Abstract

In this paper we analyze the combination of HbAS disease and haemophilia A must be exceedingly rare. Because of this rarity we report the case of two brothers with sickle cell trait and major haemophilia A. We conclude that it is about a post-circumcision bleeding due to major hemophilia A associated to sickle cell AS, this association was a systematic discovery.

## Introduction

Haemophilia A is an inherited bleeding disorder, inherited in an X-linked recessive pattern, responsible of a deficiency in anti-haemophilic factor A (factor VIII), with an incidence of 1 in 10.000 males in the world [1,2]. Sickle cell disease is a genetic disorder of hemoglobin that is transmitted as an autosomal recessive disease. It is one of the most common hereditary diseases in the world, affecting black populations especially in tropical Africa. It affects 20-25 million people globally, with high child mortality worldwide (50-80% of infants born with SCD in Africa die before the age of 5 years) [3, 4]. Sickle cell trait is the heterozygous form of sickle cell disease, it does not display the severe symptoms of sickle-cell disease [3, 4]. Combination (the coexistence) of Hb AS disease and haemophilia A must be exceedingly rare, while one is an X-linked disorder and the other a chromosomal disorder. Because of this rarity, we report the case of two brothers with concomitant Hb AS disease and major haemophilia A.

## Patient and observation

M.F. 1 year, and M.B. 2 years and half, with no particular antecedents are received in pediatric emergencies for post-circumcision bleeding. No hemostasis assessment being carried out before the act. On admission, the brothers were conscious and had a good condition. There were no familial antecedents. After stopping bleeding, blood test was requested for both brothers. For M.F: as result of hemostatic balance sheet, we found : PT= 95%, APTT= 80 sec, F VIII < 1%, F IX = 54%. Haemogram is as follow: HGB = 8.7 g/dl, MCV = 51 fl, MCH = 16 pg, WBC = 7760 /mm^3^ and platelets = 391.000/ mm^3^. Because of the microcytosis, ferritin blood test was made which was normal, about 30 ng/ml. Electrophoresis of hemoglobin was realized to explore the microcytosis, the result was : Hb A1= 60,9 %, Hb S = 35,9%, Hb A2 = 3.2%. For M.B, The hemostatic balance sheet is as follows: PT = 110 %, APTT = 72 sec, F VIII < 1%, F IX = 60 %. Haemogram : HGB = 10.7 g/dl, MCV = 66 fl, MCH = 22 pg, WBC = 6870 /mm^3^ , platelets = 193.000/ mm^3^. Ferritin was 32 ng/ml. Electrophoresis of hemoglobin was as follow: Hb A1= 57 %, Hb S = 38.5 %, Hb A2 = 4.5 %. None of the children has received a blood transfusion. We conclude that it is about a post-circumcision bleeding due to major hemophilia A associated to sickle cell AS, this association was a systematic discovery. There was no family history of sickle cell anemia, but electrophoresis of hemoglobin was realized for the mother, result was as follow: Hb A1= 62 %, Hb S = 35,5 %, Hb A2 = 2,2 %.

## Discussion

Haemophilia is a rare bleeding disorders, usually inherited, and (as it is X-linked disease) only occurring in males. There are two types of hemophilia: hemophilia A (clotting factor VIII deficiency) and hemophilia B (clotting factor IX deficiency). The incidence of hemophilia A is 4-6 times higher than of hemophilia B. Hemophilia A is characterized by a greater FVIII level less than 5% [1, 2]. Sickle cell disease (SCD) and Sickle cell trait (SCT ) are an autosomal recessive disorder involving short arm of chromosome 11. The gene defect is a known mutation of a single nucleotide of the globin gene of the hemoglobin, which results in glutamate being substituted by valine at position 6 [3, 4]. This mutation affects the syntheses of hemoglobin beta chain with formation of abnormal hemoglobin (HbS), responsible for microcirculation obstruction, ischemia, tissue necrosis and systemic organ dysfunction in the homozygote form SCD [3, 4]. Sickle cell trait (SCT) is the heterozygous state for the sickle ß-globin gene, inherited from only one parent with a phenotype Hb AS: Hb S (20-40%) and Hb A (60-80%). Individuals with SCT affected have normal life expectancy. It is usually an asymptomatic carrier without severe symptoms of SCD [3, 4]. More, The SCT confers survival advantage by providing resistance against severe malaria in the tropics. The incidence of the SCT is from 20% to 30% in Cameroon, the Democratic Republic of Congo, Gabon, Ghana, and Nigeria ranges [3, 4]. The coexistence of SCD or SCT and haemophilia is an uncommon association ant it is rarely described in literature, even in predominantly black populations, in which the prevalence of both conditions is higher, probably due to the small number of cases. We have found few associations of sickle cell disease with hemophilia; Glenn and all has reported in the literature the concurrence of hemophilia A and homozygous sickle Hb SS cell disease in a 30-year-old black male [5].

The second case was a combination of sickle cell disease Hb SS and hemophilia B, it was described in a 15-year-old African-American male [6]. Another case was a coinheritance of sickle cellβthalassemia and hemophilia A in a 19-year-old male in India [7]. The concurrence of sickle disease and hemophilia is provocative regarding possible disease interactions and modulations. SCD presents a phenotypic heterogeneity that has not been fully elucidated yet. The genetic modulators of SCD already known do not explain all the phenotypic variations. Also, no studies reported the levels of factor XIII in sickle disease and hence it is difficult to know the interaction of the two diseases. To the best of our knowledge, the relationship between SCT and haemophilia has not been elucidated yet. In another way, both SCT and SCD are strongly associated with a high risk of venous thrombo-embolism, as variously reported in the literature. In fact, many studies demonstrated that persons with SCT had an increased risk of thrombotic accidents such as pulmonary embolism and DVT, in the absence of any other known prothrombotic risk factors. These studies strongly suggest that the sickling disorders are essentially thrombophilic [8-10]. This due to the combined hypercoagulable effects of red cell sickling and the release of procoagulant red cell membrane phospholipids. Moreover, both SCD and SCT are associated with relative elevation of monocyte count whih is increased expression of monocyte-derived tissue factor, and aggravate the prothrombotic hypercoagulability [11, 12].

Many authors considering that SCT as an hypercoagulable prothrombotic state and hypothesise that coinheritance of the either SCT or SCD with hemophilia would be expected to modify the haemophilic thrombo haemorrhagic balance and should positively ameliorate haemophilic bleeding phenotypes [13]. This hypotheses will be particularly interesting in severe haemophilia with SCT, where patients could have lower frequencies of spontaneous bleeding episodes than those with normal Hb phenotype (Hb AA phenotype). However, very few reports in the literature suggest this hypothesis. We found a case report of a patient from India, which coinheritance of severe haemophilia A and SCD, revealed lower incidence of bleeding complications [7]. A retrospective study from Nigeria, revealed lower frequencies of spontaneous bleeding in patients with both severe haemophilia-A and SCT [13]. In our case, the results of the mother shows that both diseases were inherited from her, the father is not reached of heamophilia. Both the two patients had a factor VIII level of <1%, no spontaneous hemorrhagic accident was revealed in the past, the first hemorrhagic incident was due to a chirurgical act (circumcision), but the earlier age of our patients (1 and 2 years) don't allow us to confirm that sickle cell disorder protects from spontaneous bleeding.

## Conclusion

The combination of two monogenic diseases in one individual patient leaves us perplexed. The limited number of cases published in the literature on the coexistence of hemophilia and SCD or SCT in the same individual does not allow stating if patients with sickle cell disorders are more likely to develop haemophilia or the reverse. This therefore deserves to be mentioned, in order to launch in-depth genetic and molecular studies to unravel the mystery. Almost, the coinheritance of a bleeding disorder with a hypercoagulable state prompted us to report this two cases, in order to give attention to this conc rarely described.

## Competing interests

The author declare no competing interests.
